# Prevalence of diffuse idiopathic skeletal hyperostosis (DISH) assessed with whole-spine computed tomography in 1479 subjects

**DOI:** 10.1186/s12891-018-2108-5

**Published:** 2018-05-30

**Authors:** Akihiko Hiyama, Hiroyuki Katoh, Daisuke Sakai, Masato Sato, Masahiro Tanaka, Masahiko Watanabe

**Affiliations:** Department of Orthopaedic Surgery, Tokai University School of Medicine, 143 Shimokasuya, Isehara, Kanagawa 259-1193 Japan

**Keywords:** Diffuse idiopathic skeletal hyperostosis (DISH), Computed tomography (CT), Prevalence, Thoracic spine

## Abstract

**Background:**

Computed tomography (CT) analyses have reported that the prevalence of diffuse idiopathic skeletal hyperostosis (DISH) in Japan is 8.7–27.1%. However, these data were obtained using chest-abdominal CT, and no evaluations of sagittal, coronal, and axial images using whole-spine CT have been reported. The aim of this study was to investigate the prevalence and characteristic of DISH by whole spinal CT.

**Methods:**

Participants were patients who had experienced trauma who had undergone whole-spine CT scanning based on the initial clinical practice guidelines for trauma in our institute from April 2015 to February 2018. The subjects were > 20 years old and 1479 were included in the analysis. The presence and distribution of DISH and clinical parameters such as age and sex were reviewed retrospectively according to the location of DISH.

**Results:**

The overall prevalence of DISH was 19.5% (*n* = 289). Subjects with DISH were older than those without. DISH was located in the thoracic spine in 65.1% and thoracolumbar spine in 24.2% of patients. More than 80% of ligamentous ossifications associated with DISH occurred at T8 (*n* = 255, 88%), T9 (*n* = 262, 91%), and T10 (*n* = 247, 85%). Most of the ossification occurred to the right anterior of the vertebral body, and there were few ossifications in the areas in contact with the artery and vein.

**Conclusions:**

The prevalence of DISH based on whole-spine CT was 19.5%. Ossification was noted more often at T8, T9, and T10, and to the right anterior of the vertebral body. It is for the first time report that we have studied the location of ossification in detail using the axial images of whole spine CT. We hope this study will enhance the understanding of the characteristics of DISH.

**Electronic supplementary material:**

The online version of this article (10.1186/s12891-018-2108-5) contains supplementary material, which is available to authorized users.

## Background

Recently, much attention has focused on vertebral fractures accompanying diffuse idiopathic skeletal hyperostosis (DISH). That is, an ankylosed spine tends to fracture as result of minor injuries such as falling because of stress concentration. Such fractures can cause reverse chance fractures that can lead to spinal paralyses, and are often resistant to conservative treatment [1–3]. Like other conditions with ligament ossification, DISH is thought to result from a multifactorial process whose etiology is not easily identified. In Japan, which is experiencing an aging society, the prevalence of minor paralysis caused by trauma is on the rise.

Proposed by Resnick et al. in 1975, DISH is a degenerative disease in which the spinal longitudinal ligaments and entheses gradually become ossified [4]. The pathogenetic mechanisms responsible for DISH are poorly understood, but genetic, metabolic, endocrine, anatomic, environmental, and toxic factors may contribute to its’ development [5, 6]. Moreover, DISH is a condition of the elderly and is rarely seen before middle age. It is more common in males than in females with male/female ratios ranging between 2:1 and 7:1 [7, 8]. The criteria most often used to diagnose DISH was first reported by Resnick and Niwayama in 1976 [9], before computed tomography (CT) was available as a diagnostic tool. However, when examining the prevalence of DISH, the problem is that the criteria also differs according to the article [9, 10].

The prevalence of DISH reported both in Japan and internationally varies widely between 3.8–25% [8, 9, 11–15]. It is considered to occur more frequently in Caucasians than in people of Asian and Black ethnicity. However, some groups have suggested that this considerable variation could be attributed to the incomplete diagnosis of DISH based on X-ray alone. CT provides far more detailed imaging of the intervertebral disc spaces and bridging ossifications, but there are only a few reports on CT-based diagnosis of DISH. Analyses using CT have reported that the prevalence of DISH in Japan averages 8.7% (13% for males, 2.5% for females) and may be as high as 27.1% (38.7% for males, 13.9% for females) [12, 16]. However, these data were obtained using chest-abdominal CT, and evaluations using sagittal, coronal, and axial whole-spine CT have not been reported. Most of the patients surveyed so far have been older than 40 years and, therefore, the true prevalence is unknown. The purpose of this study was to use whole-spine CT to investigate the prevalence and characteristics of DISH in trauma patients examined in an emergency critical care setting. In addition, we hope this study will enhance the understanding of the characteristics of DISH.

## Methods

Our institution is a tertiary emergency medical facility that treats over 800 patients with severe trauma a year. The patients included in this study were those who had undergone whole-spine CT scanning during the period between April 2015 to February 2018 based on the clinical practice guidelines for trauma patients at our institute. The patients were aged over 20 years and their mean age was 54.7 years.

A total of 1479 subjects were included in the analysis: 1023 males and 456 females. The presence and distribution of DISH, and their clinical parameters such as age and sex were reviewed retrospectively and classified according to the location of DISH. The numbers of subjects in the age groups 20–39, 40–49, 50–59, 60–69, 70–79, 80–89, and ≥90 years were described in Table 1.

**Table 1:** Distribution of study population according to sex

Age group, yrs	Male	Female	ALL
20–39	286	93	379
40–49	167	60	227
50–59	160	46	206
60–69	167	70	237
70–79	162	98	260
80–89	70	77	147
90-	11	12	23
	1023	456	1479

### Computed tomography (CT) examination

**Fig. 1:**
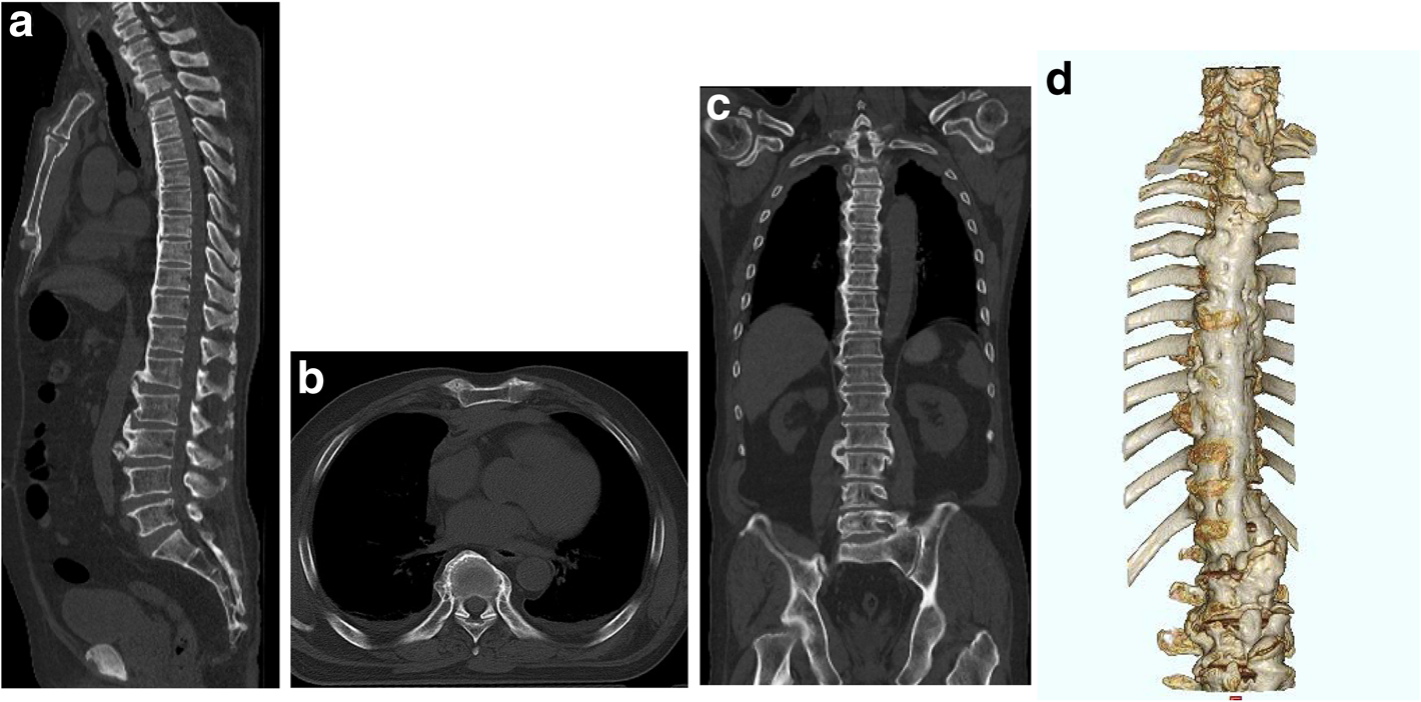
CT images of a patient with DISH. Reconstructed CT of sagittal (**a**), axial (**b**), coronal (**c**) and 3D (**d**) views in a patient with DISH

All CT scans were performed on a multidetector CT (120 kV, 380 mA, 0.6 mm slice; SOMATOM Definition AS; Siemens Healthcare, Forchheim, Germany) equipped with a 128-slice multidetector array. The diagnosis of DISH was based on whole-spine CT according to the criteria proposed by Resnick and Niwayama [9]: flowing ligamentous ossification in ≥4 contiguous vertebrae of the spine, with preserved intervertebral disc space, and the absence of inflammatory changes in the apophyseal joints or the sacroiliac region. The reconstructed CT sagittal, coronal, and axial images (Fig. 1) were used rather than X-ray to evaluate the continuous ligament ossification. All CT data were evaluated by authors. For testing the reliability of diagnoses, the testers read the same images of 20 patients to check inter-observer agreement. We also used intra-observer error at different time points, and the interval was found to be longer than one month by the first author. Differences were settled by consensus to minimize intra- and inter-observer bias and errors. Finally all images were reviewed by the first author.

### Statistical analysis

Statistical analysis was performed using SPSS software for Windows (v. 20.0; IBM Corp., Armonk, NY, USA). All values are expressed as mean ± standard deviation. We studied statistical differences between the group with and without DISH. Analysis of DISH prevalence in males and females was performed using the chi-squared test. Analysis of variance with a post hoc test (Mann–Whitney *U* test) was used for comparisons. *P*-values < 0.05 were considered significant.

## Results

The Kappa coefficient of inter- and intra-observer agreements were 0.79 and 0.89, respectively.

**Fig. 2:**
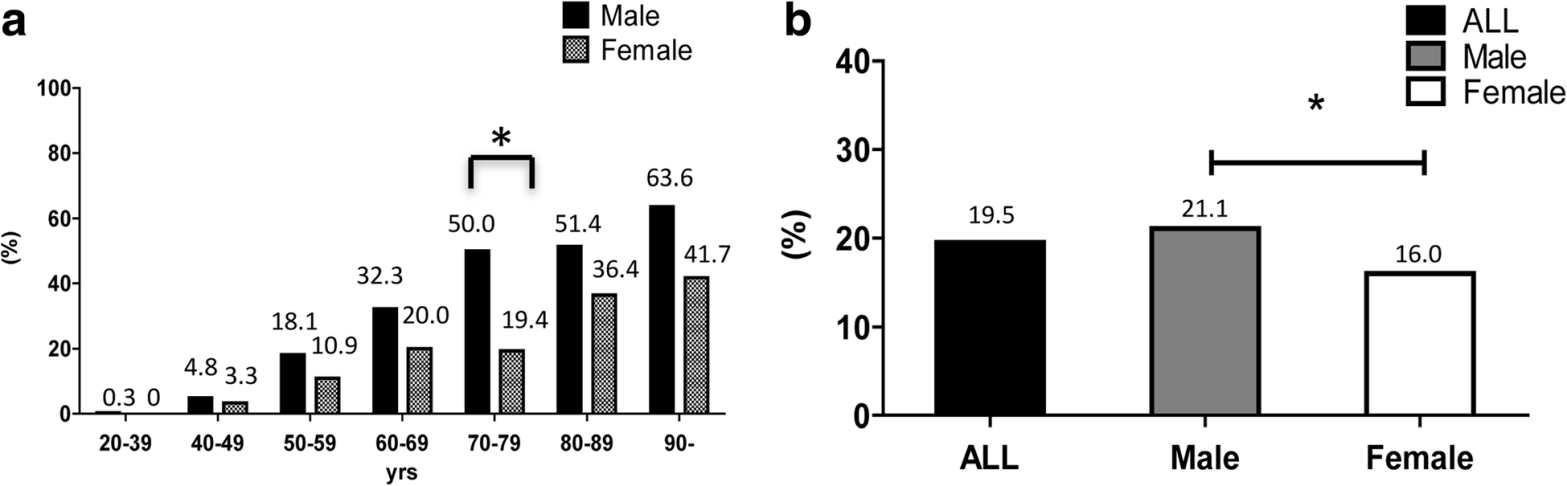
Prevalence of DISH detected by CT. **a** Age distribution of the prevalence of DISH detected by CT. The prevalence of DISH increased with age in both males and females. **b** Comparison of the prevalence of DISH between male and female

The demographics of the subjects included in this study are shown in Table 2. The prevalence of DISH was 19.5% (289/1479). The subjects with DISH were significantly older than those without: 71.7 and 50.6 years, respectively (*P* < 0.001). The prevalence of DISH increased with age. The prevalence of DISH in subjects aged ≥70 years was 40.9% (176/430), indicating that one in two people ≥70 years had DISH. In addition, the prevalence of DISH increased with age in both males and females (Fig. 2a). The results showed that the prevalence rates classified by age groups 20–39, 40–49, 50–59, 60–69, 70–79, 80–89, and ≥90 years were 0.3, 4.8, 18.1, 32.3, 50.0, 51.4, and 63.6% in male and 0, 3.3, 10.9, 20.0, 19.4, 36.4, and 41.7% in female, respectively. There were a significantly greater number of subjects with DISH in their 70s using the chi-squared test.

**Table 2:** Distribution of study population according to DISH(+) or (−)

	DISH (+)	DISH (−)	ALL
Sex	N	N	N (%)
Male	216	807	1023 (69.2)
Female	73	383	456 (30.8)
Male to Female	3.0: 1	2.1: 1	2.3: 1
Age, Mean	71.7	50.6	54.7
Age group, yrs	N (%)	N (%)	N
20–39	1 (0.3)	378 (99.7)	379
40–49	10 (4.4)	217 (95.6)	227
50–59	34 (16.5)	172 (83.5)	206
60–69	68 (28.7)	169 (71.3)	237
70–79	100 (38.5)	160 (61.5)	260
80–89	64 (43.5)	83 (56.5)	147
90-	12 (52.2)	11 (47.8)	23
Total (%)	289 (19.5)	1190 (80.5)	1479

*DISH* diffuse idiopathic skeletal hyperostosis, *N* number

Among the 289 subjects with DISH, 216 (prevalence 216/1023 = 21.1%) were male and 73 (73/456 = 16.0%) were female (Fig. 2b). This tendency for a higher frequency in male was a significant (*P* < 0.05). In addition, when analyzed with subjects ≥40 years as in previous reports, the prevalence of DISH in 737 males and 363 females was 29.2% (215/737) and 20.1% (73/363), respectively, also showing a significant sex difference (*P* < 0.01) (data not shown).

**Fig. 3:**
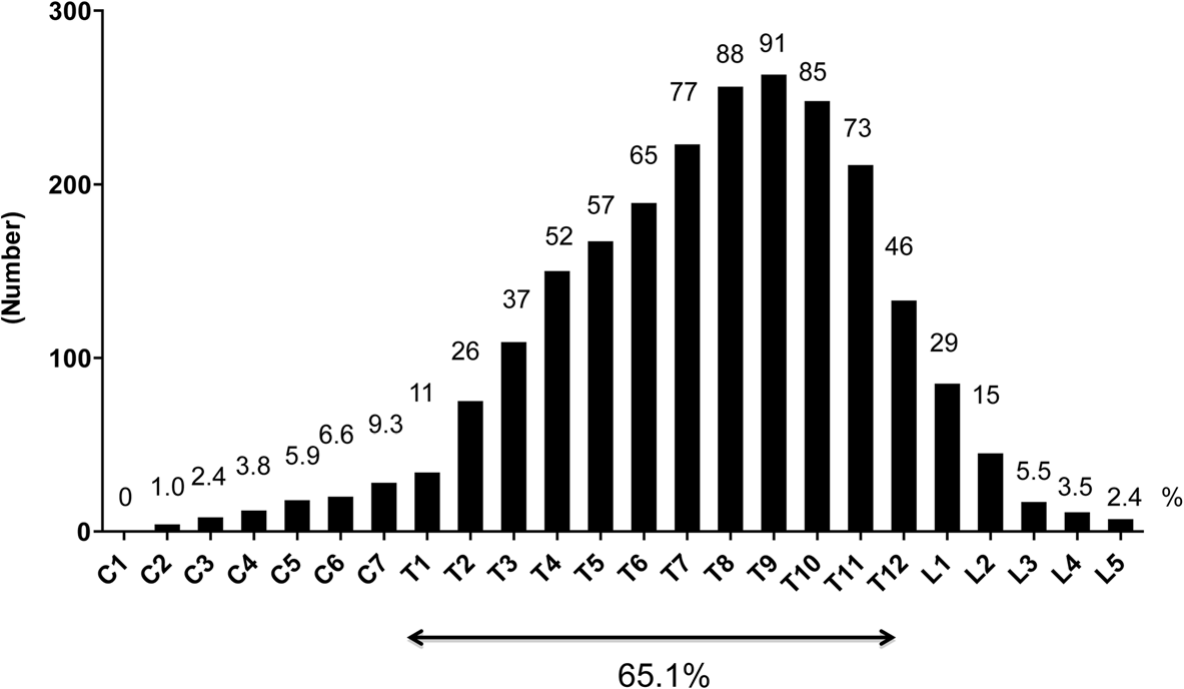
Prevalence of DISH classified by location in the spine. The levels of fused segments are shown from the cervical to lumbar spine, and involved mainly T8–10

We classified DISH into six types according to location in which ossification along the aspect of ≥4 contiguous vertebral bodies was observed: (1) cervical—ossification only in the cervical region (C1–C7); (2) cervicothoracic—ossification only in the cervicothoracic region (C1–T12); (3) thoracic—ossification only in the thoracic region (T1–T12); (4) thoracolumbar—ossification only in the thoracolumbar region (T1–L5); (5) lumbar—ossification only in the lumbar region (L1–L5); and (6) whole spine—ossification in the entire spine (C1–L5). Figure 3 shows the prevalence of DISH classified by location in the spine: 65.1% were thoracic, and 24.2% were thoracolumbar. The prevalence of cervical and lumbar spine involvement was low.

Evaluation of the fused segments from the cervical to lumbar spine for all subjects revealed that the eighth thoracic (T8) to the 10th thoracic (T10) levels were the levels most involved, with more than 80% of the ligamentous ossifications associated with DISH located at T8 (*n* = 255segments/289subjects, 88%), T9 (*n* = 262segments/289subjects, 91%), or T10 (*n* = 247segments/289subjects, 85%) (Fig. 3). Of the 233 cases with consecutive ossification at T8–T10, the apex of thoracic kyphosis was located at T8–T10 in 142 subjects (60.9%) (Table 3).

**Table 3:** Among 233 subjects who had consecutive ossification in T8 - T10, 142 subjects were found at the apex of the thoracic kyphosis at T8 - T10

Apex of kyphosis	N	%	
T5	1	0.4	
T6	11	4.7	
T7	37	15.9	
T8	68	29.2	
T9	47	20.2	142 (60.9%)
T10	27	11.6	
T11	25	10.7	
T12	11	4.7	
L1	5	2.1	
L3	1	0.4	
	233		

*N* number

**Fig. 4:**
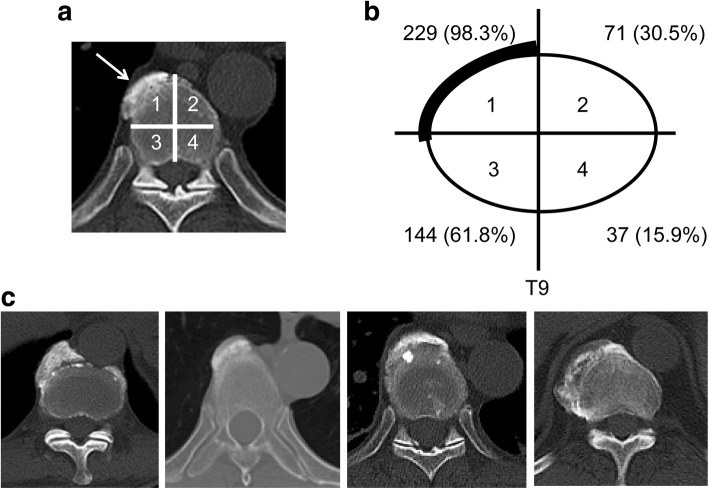
Axial (AX) image of the areas showing ossification at T9. **a** A 58-year-old man presented with DISH between T7–11. The vertebral body was divided into 4 areas from the AX image of T9. In this case ossification was observed in area 1 (arrow). **b** Location of ossification on vertebral body: Area 1, right anterior; Area 2, left anterior; Area 3, right posterior; Area 4, left posterior. **c** Ossification was rarely found in vertebral bodies contacting with the aorta and the azygous vein

Axial images of the ossification lesions at T9 (Fig. 4a) were studied further focusing on the location of the ossification, revealing that the ossification at T9 was observed in almost all cases in area 1, which is the right anterior region of the vertebra (98.3%) (Fig. 4b). As age increased, ossification in the left anterior region also appeared (Table 4). In a few subjects, the ossification was observed in the area in contact with the aorta and the azygous vein (Fig. 4c). The average number of vertebral bodies continuously bridged by ossification of the anterior longitudinal ligament was 7.9, and involvement of ≥12 vertebrae was seen in 47 of the 289 patients (16.3%). The average numbers of ossified vertebrae according to the age groups 20–49 (*n* = 11), 50–59 (*n* = 34), 60–69 (*n* = 68), 70–79 (*n* = 100), 80–89 (*n* = 64), and ≥90 (*n* = 12) years were 6.8 ± 2.0, 6.8 ± 2.6, 7.4 ± 3.2, 8.3 ± 3.5, 8.3 ± 4.2, and 9.4 ± 3.6, respectively, showing that the number of vertebrae with ligamentous ossification increased with age.

**Table 4:** Axial image of the lesion of ossification at the T9 level among 233 subjects who had consecutive ossification in T8 - T10

		T9 Lesion				T9 Lesion			
		1	2	3	4	1	2	3	4
Age, yrs		N				%			
20–39	1	1	0	0	0	100	0	0	0
40–49	7	6	1	3	1	85.7	14.3	42.9	14.3
50–59	24	24	3	18	1	100.0	12.5	75.0	4.2
60–69	54	52	6	24	3	96.3	11.1	44.4	5.6
70–79	86	85	25	56	10	98.8	29.1	65.1	11.6
80–89	51	51	29	35	18	100.0	56.9	68.6	35.3
90-	10	10	7	8	4	100.0	70.0	80.0	40.0
	233	229	71	144	37	98.3	30.5	61.8	15.9

*N* number

## Discussion

DISH is a common condition in the elderly, but DISH is still insufficiently investigated and understood. In this study, CT images of the whole spine were used to determine the prevalence of DISH in 1479 patients aged > 20 years. While the prevalence of DISH has been reported to vary widely from 3.8 to 25%, it was 19.5% in these subjects. Factors that may affect the prevalence of DISH include differences in ethnic and genetic background, diagnostic imaging methods, patient age, and lifestyle habits. The prevalence of DISH in Japan, which would exclude the effects of race and genetic factors, has been reported to range from 8.7 to 27.1% [12, 13, 16]. Based on chest X-rays of 1363 patients, Weinfeld et al. showed that the prevalence differed between ethnic groups [14]. They reported DISH to be less common in the black, Native-American and Asian populations.

The prevalence of DISH may differ between past and present reports because of advances in diagnostic imaging (Additional file 1). In past reports, DISH was identified by X-ray alone, which resulted in lower prevalence than that identified by recent reports of DISH identified through examination of chest CT. Hirasawa et al. compared the prevalence of DISH evaluated by reconstructed CT of the chest to pelvis against plain radiography of the chest and abdomen [12], and found that the prevalence of DISH based on CT was 27.1% and that based on X-ray was 17.6%. Mori et al. reported that the prevalence of DISH was 8.7% using chest CT performed for investigation of pulmonary disease [16], which was smaller than that reported by Hirasawa et al. [12]. The differences in prevalence between these studies may relate to the sample size: that Mori et al. evaluated a large number of subjects (3013 cases), but was a patient-based and not a population-based study [16]. They acknowledged that the relationship between pulmonary disease and ossification of the spinal ligaments, including DISH, could have affected the prevalence data. Moreover, the study by Mori et al. included only chest CT and was not based on CT of the chest to pelvis, which may account for the differences from the study by Hirasawa et al. [12]. Our findings and those of previous reports indicate that most cases of DISH occur in the middle and lower thoracic spine. This means that when making the diagnosis, evaluation using whole-spine CT will be more effective than X-ray [12, 16]. On the accuracy of DISH diagnosis by CT, intra- and inter-observer error by review of CT was also less than that of X-ray [12]. From these data, we think that CT may be a most suitable modality for the evaluation of DISH. Thus, analysis of the prevalence of DISH and its characteristics from the analysis using CT is considered to be useful for analyzing the treatment and pathology.

Another possible reason for the difference in prevalence of DISH is age of the study population. Most previous reports only included people older than 40 years with an average of 65 years [8, 13, 17], whereas in this study, we included younger patients (> 20 years) for an average age of 54.7 years old.

One limitation of our study is that the patients’ past history was not analyzed; in particular, the presence of metabolic disease, because the distribution of these patients may affect the prevalence of DISH. Although the pathogenesis of DISH has not been fully elucidated, DISH may be related to modern lifestyle-related diseases such as obesity and metabolic syndrome, and the prevalence rate of DISH may be higher in those with metabolic disease. Some have suggested that its prevalence may increase in the coming decades because of the relationship between DISH and such modern lifestyle-related diseases [5, 6, 18, 19]. We could not investigate any possible associations between such factors and DISH because no clinical background information except for age and sex were available in our retrospective study.

Other studies have reported a higher prevalence of DISH in males than in females. The Research on Osteoarthritis/Osteoporosis Against Disability (ROAD) study of Kagotani et al. investigated the prevalence of DISH in 1647 people who underwent whole-spine X-ray [13]. Their logistic regression analysis revealed that the prevalence of DISH was associated with male sex (odds ratio (OR): 5.55) and presence of severe lumbar spondylosis (OR: 5.50). It appears that male tend to be more likely to develop DISH than female regardless of ethnicity or genetic factors. In our study, there was a tendency for the prevalence of DISH to be higher in males than in females, but the difference was small than in previous studies. It is possible that this result may have been affected by the inclusion of younger patients than in previous studies.

The most common site of DISH is the thoracic spine. We found that most of the ossification occurred in the middle and lower thoracic spine: T8 (88%), T9 (91%), and T10 (85%). It is interesting that DISH occurs at these sites. Hirasawa et al. and Kagotani et al. also found that DISH frequently occurred at T7–10 [12, 13]. Hirasawa et al. reported that more than 80% of DISH occurred at T8/9 or T9/10, which may reflect an anatomical effect; these vertebrae are susceptible to compressive mechanical stress because T8 is located almost at the peak of the physiological spinal kyphosis. Accordingly, DISH seems to arise mainly from the thoracic spine and may extend to the cervical and/or lumbar spine because of mechanical stress. Similar results were obtained in this study when the peak of spinal kyphosis was evaluated by supine CT. Interestingly, most of the ossification occurred to the right anterior of the vertebral body, and there were few ossifications in the area in contact with the arteries and veins. Previous study also demonstrated that the location of ossification was termed as anteolateral or right-sided hyperostosis [10]. Arterial blood flow and blood pressure may be affecting the ossification progress of DISH. Unfortunately we do not know about this mechanism from this study. However, it is for the first time report that we have studied the location of ossification in detail using the axial images of whole spine CT.

To date, the current knowledge on the pathogenesis of DISH is very limited. Some groups have reported that the pathogenesis of DISH is based on the excess of growth factors that might induce transformation of mesenchymal cells into fibroblasts and osteoblasts [20–22]. We think that it is need further analysis of these molecular mechanisms, which is important to clarify the etiology of DISH in the future. Okada et al. reported that the thoracolumbar junction (T11–L2) was the most frequently fractured level (54.8%) in patients with DISH [1]. The fact that vertebral body fractures associated with DISH are more frequent in the thoracolumbar junction may be related to the fact that most of DISH occurs from T8 to T10. However, in this cross-sectional study, it was impossible to evaluate whether DISH occurred first in the thoracic spine and then secondarily in the cervical or lumbar spine.

This study has some limitations. First, we did not have access to clinical data such as the presence of diabetes, which is a risk factor associated with DISH. This was a retrospective study and it was not possible to investigate all subjects. Second, there may have been selection bias in the inclusion of subjects due to the fact that they were all trauma patients, but we did not have access to data about each patient before their trauma. DISH patients are usually asymptomatic, and health checks of the general population will be an excellent way to evaluate the prevalence of DISH. Finally, the radiation exposure experienced in whole-spine CT imaging in normal subjects is a health risk that must be considered. Some researchers agree that, when used correctly, such scans can save lives. However, according to some estimates, the radiation exposure a patient receives from a full-body CT scan is often 500 times that of an X-ray. Despite these limitations, our data suggest that the prevalence of DISH in Japan is larger than in past reports. We believe that prospective multicenter studies are needed to determine the prevalence and pathogenesis of DISH.

## Conclusions

The prevalence of DISH based on whole-spine CT was 19.5%, which is similar to that reported by other studies. Most ossification occurred at levels T8–10 and in the right anterior region of the vertebral body.

## Additional file

Additional file 1: Previous of DISH in each investigative modality. N: number, Xp: x-ray, AP: anterior-posterior, PA: posterior-anterior, CT: computed tomography. (DOC 47 kb)

## Abbreviations

CT: Computed tomography
DISH: Diffuse idiopathic skeletal hyperostosis
ROAD: Research on Osteoarthritis/Osteoporosis Against Disability
