# Supplementary material for: Prevalence of diffuse idiopathic skeletal hyperostosis (DISH) assessed with whole-spine computed tomography in 1479 subjects
Source: BMC Musculoskelet Disord. 2018 May 30;19:178. doi: 10.1186/s12891-018-2108-5 (PMC5977547; doi:10.1186/s12891-018-2108-5)
Supplement: Supplementary file 1 — Previous of DISH in each investigative modality. N: number, Xp: x-ray, AP: anterior-posterior, PA: posterior-anterior, CT: computed tomography. (DOC 47 kb) [file 12891_2018_2108_MOESM1_ESM.doc]

Additional file 1

| **Authors** | **Year** | **N** | **Prevalence%** | **Average Age** |  | **Modality** | **Race** |
| --- | --- | --- | --- | --- | --- | --- | --- |
|  |  |  | **ALL** |  |  |  |  |
| Julkunen | 1975 | 8993 | 2.6 | - | >40 | Chest Xp(L) | Finland |
| Resnick | 1976 | 215 | - | 75 | >46 | Autopsy | USA |
| Boachie-Adjei | 1987 | 75 | 28 | 65 | >50 | Autopsy/ChestXp(AP/L) | USA |
| Cassim | 1990 | 1500 | 3.9 | - | >40 | Chest Xp(L) | South Africa |
| Weinfeld | 1997 | 2364 | - | - | >50 | Chest Xp(PA/L) | USA |
| Kiss | 2002 | 635 | 3.6 | - | >50 | Chest Xp(PA/L)lumbar Xp | Hungary |
| Kiss | 2002 | 635 | - | - | >50 | Chest Xp(PA/L), lumbar Xp | Hungary |
| Kim | 2004 | 3595 | 2.9 | 64.25 | >50 | Chest Xp(L) | Korea |
| Westeveld | 2008 | 501 | 17 | 66.6 | >50 | Chest Xp(PA/L) | Netherland |
| Holton | 2011 | 298 | 42 | 74 | >65 | Spine (T2-S1)Xp(L) | USA |
| Diederichs | 2011 | 342 | 38(52) | 74.2 | >65 | T-lumbar Xp(L) | USA |
| Kagotani | 2014 | 1647 | 11 | 65.3 | >23 | Whole SpineXp(AP/L) | Japan |
| Nardo | 2014 | 1172 | 13 | - | 70-79 | Spine CT | USA |
| Hirasawa | 2016 | 558 | 27.1(CT) | 66.7 | >40 | chest-Abd CT,T-AbdoXp(AP) | Japan |
|  |  |  | 17.6(Xp) |  |  |  |  |
| Mori | 2017 | 3013 | 8.7 | 65 | >16 | Chest CT | Japan |
| Katzman | 2017 | 1591 | 13.5 | 71.5 | >50 | T-lumbar Xp(L) | USA |
| Fujimori | 2017 | 1500 | 12 | 57 | >21 | Whole Spine PETCT | Japan |
| Toyoda | 2017 | 345 | 25.6 | 63.5 | >20 | Whole spineXp | Japan |
| Bateman | 2018 | 459 | 30 | 68 | >50 | Chest Xp | Pacific Islander |
